# Endovascular Management of Traumatic Vertebral Artery Pseudoaneurysm Associated with Vertebral Arteriovenous Fistula Using a Covered Stent

**DOI:** 10.7759/cureus.5716

**Published:** 2019-09-21

**Authors:** Arun Gupta, Vivek Murumkar, Sameer Peer

**Affiliations:** 1 Neuroimaging and Interventional Radiology, National Institute of Mental Health and Neurosciences, Bangalore, IND; 2 Neuroimaging and Interventional Radiology, National Institute of Mental Health and Neuro Sciences, Bangalore, IND

**Keywords:** pseudo-aneurysm, arteriovenous fistula, stent

## Abstract

Vertebral arteriovenous fistula is an abnormal communication between the vertebral artery and adjacent venous structures. The most common cause of vertebral arteriovenous fistula is trauma, however, spontaneous occurrence is also known. We report a case of traumatic pseudoaneurysm with associated vertebral arteriovenous fistula which occurred following stab injury to the left vertebral artery. The diagnosis was suspected clinically and on CT angiography. Digital subtraction angiography confirmed the diagnosis, and a covered stent was placed in the left vertebral artery at the site of the injury following which there was complete closure of the pseudoaneurysm and fistula and anterograde flow was restored in the vertebral artery.

## Introduction

Vertebral arteriovenous fistula is a rare clinical entity in which a direct communication exists between the vertebral artery, its muscular or radicular branches and the surrounding venous structures [1]. The most common cause of vertebral arteriovenous fistula is blunt or penetrating trauma to the neck. Spontaneous vertebral arteriovenous fistulae are known to occur in patients with known predisposing factors e.g. neurofibromatosis I, Ehlers-Danlos syndrome and fibromuscular dysplasia [1,2]. Endovascular occlusion of the fistula is considered as the standard of care in the management of vertebral arteriovenous fistula [1,2].

## Case presentation

A 26-year-old man was brought to the emergency department of our hospital following a stab injury on the left posterolateral aspect of the neck. The patient developed a progressively enlarging swelling in the left posterolateral aspect of the neck within two days following the penetrating injury. The patient also complained of headache and giddiness following the injury. There was no neurological deficit. The patient was conscious and oriented to time, place and person. His vitals were stable. On visual inspection, a gaping wound was noted at the entry site of the penetrating injury. However, there was no active bleeding through the wound. On palpation, the swelling was pulsatile and a thrill could be felt. He did not have any comorbid illness. He did not give any history of substance abuse. His social and family history was unremarkable.

**Figure 1:**
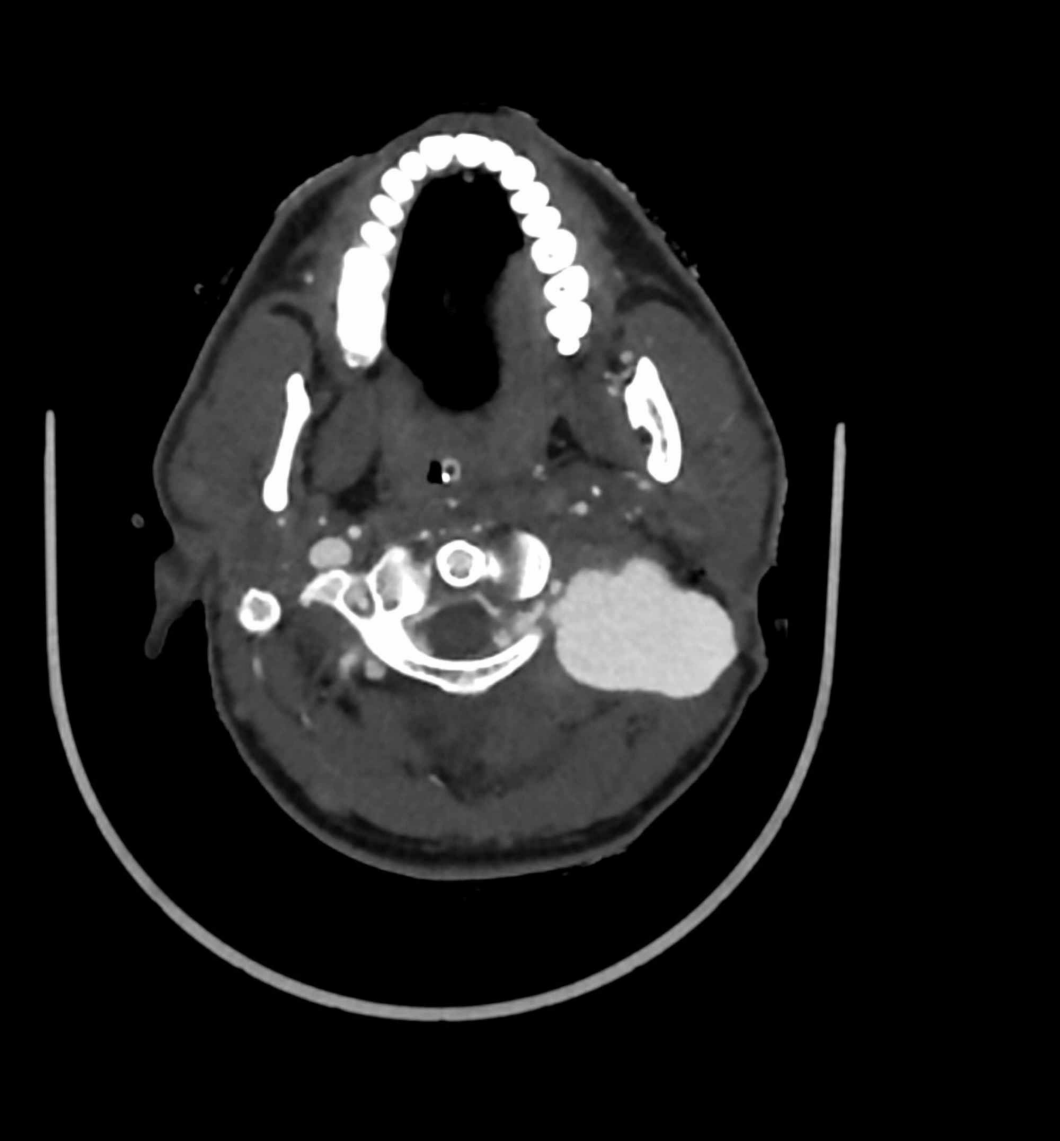
CT angiogram shows a pseudoaneurysm arising from the V2 segment of the left vertebral artery. The epidural venous plexus appears engorged which is suggestive of a vertebral arteriovenous fistula.

CT angiogram was performed which showed an enhancing sac-like structure on the left paravertebral region with apparent communication with the V2 segment of the left vertebral artery (Figure 1). The epidural venous plexus appeared engorged. A diagnosis of left vertebral artery pseudoaneurysm with arteriovenous fistula was suspected on the basis of the clinical and CT angiogram findings.

**Figure 2:**
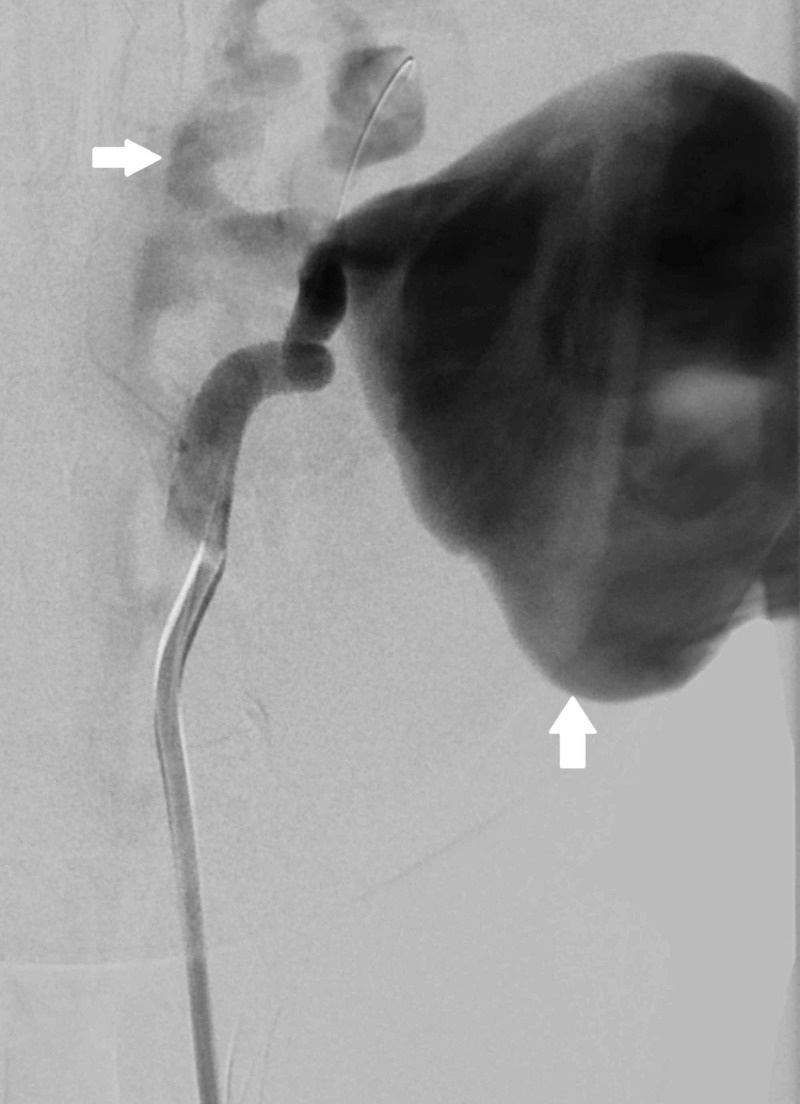
Digital subtraction angiogram with left vertebral artery injection shows filling of a large pseudoaneurysm with opacification of the vertebral venous plexus in arterial phase suggestive of a vertebral arteriovenous fistula (arrows). There is steal of contrast into the pseudoaneurysm and the fistula with no anterograde flow in left vertebral artery.

On digital subtraction angiogram, there was evidence of extravasation of the contrast through the V2 segment of left vertebral artery at the level of C2 vertebral body which was then seen to be draining into vertebral venous plexus (Figure 2). There was no anterograde flow distal to the site of fistula.

**Figure 3:**
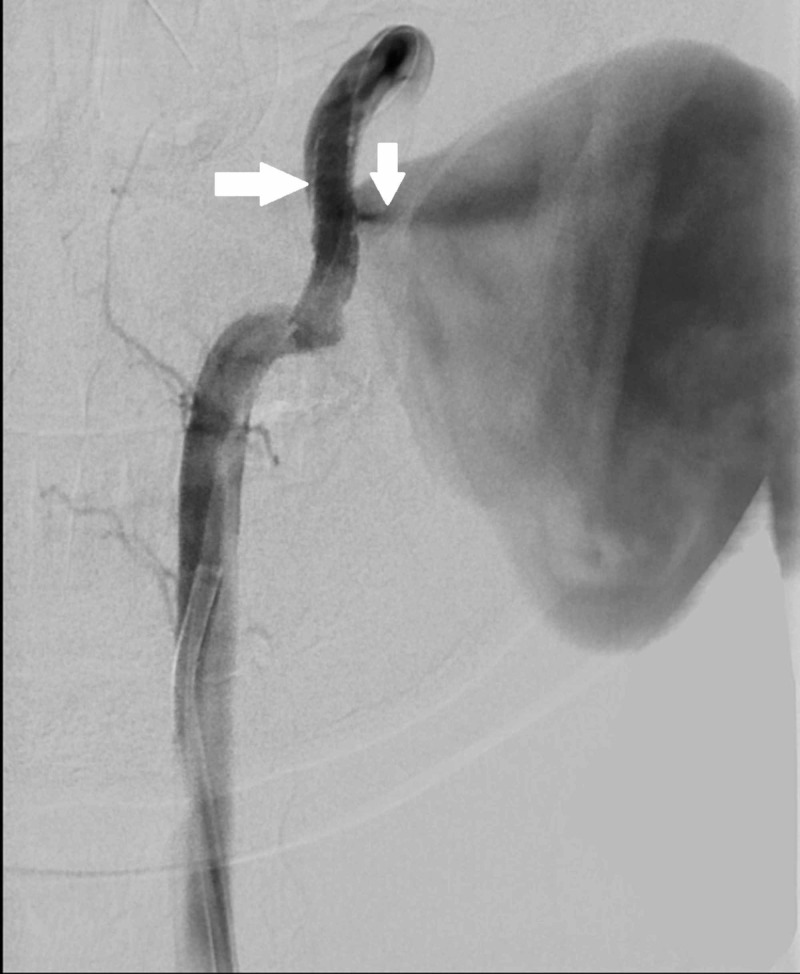
Digital subtraction angiogram with left vertebral artery injection immediately after the deployment of the stent shows filling of the pseudoaneurysm as the stent was undersized (arrows). Note that no venous filling is seen in arterial phase after deployment of the stent (as compared to Figure 1) due to reduction in blood flow into the pseudoaneurysm and the fistula.

A covered stent was deployed across the site of fistula to exclude the pseudoaneurysm from the main left vertebral artery which would also close the direct arteriovenous communication. We used a 6F guide catheter (Envoy, Codman Neurovascular, Raynham, MA) over a 0.035" guide wire (Terumo) and navigated it into the V2 segment of left vertebral artery using roadmap guidance. Following this, a 0.014” microwire (Traxcess, Microvention) was advanced through guide catheter and the site of fistula was crossed. A covered stent (Graftmaster, Abbott Vascular) of size 3.5 mm x 19 mm was taken over microwire. The stent was then deployed covering V2 and V3 segments of the left vertebral artery. A check angiogram after the deployment of stent showed residual filling of the pseudoaneurysm and the fistula (Figure 3). This was probably due to the fact that the vessel calibre was slightly larger than the size of the stent.

**Figure 4:**
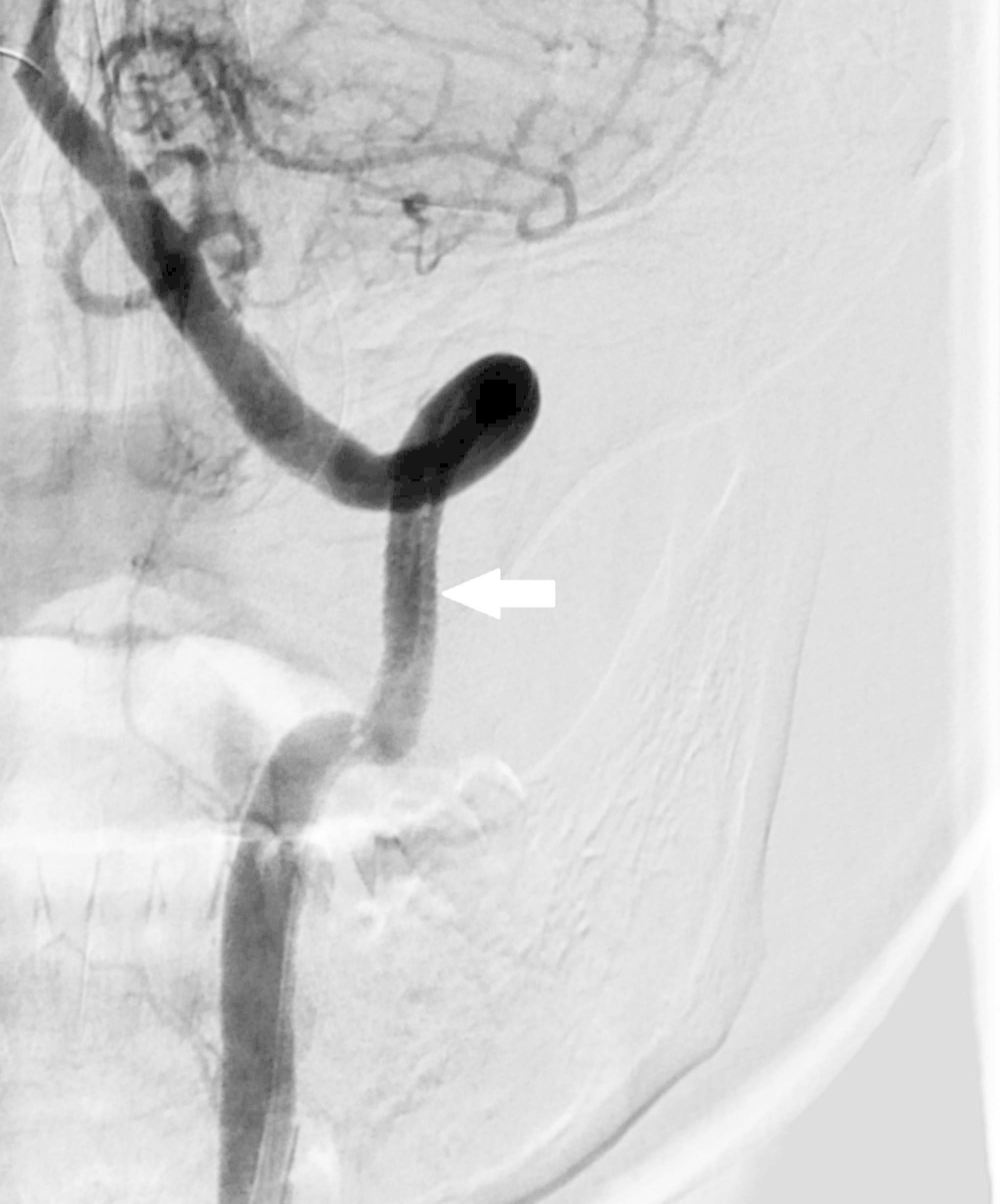
Digital subtraction angiogram with left vertebral artery injection after balloon dilation of the stent with approximation of the stent to the arterial wall shows no evidence of filling of the pseudoaneurysm and the fistula (arrow).

Then, we dilated the stent with a balloon. A 5 mm x 20 mm, 135 cm size balloon (Viatrac 14 plus, Abbott Vascular) was taken over the 0.014" wire, and the stent was dilated to approximate the vessel walls. Check angiogram showed no residual filling of the fistula with good anterograde flow in the left vertebral artery (Figure 4).

The patient was started on IV tirofiban at 28 ml/hr infusion for 30 minutes followed by 7 ml/hr for 18 hours post-procedure. A bridging dose of aspirin 300 mg orally and clopidogrel 300 mg orally was given six hours before stopping tirofiban. The patient was discharged in stable condition 24 hours after the intervention on aspirin 150 mg once a day orally and clopidogrel 75 mg once a day orally for three months.

**Figure 5:**
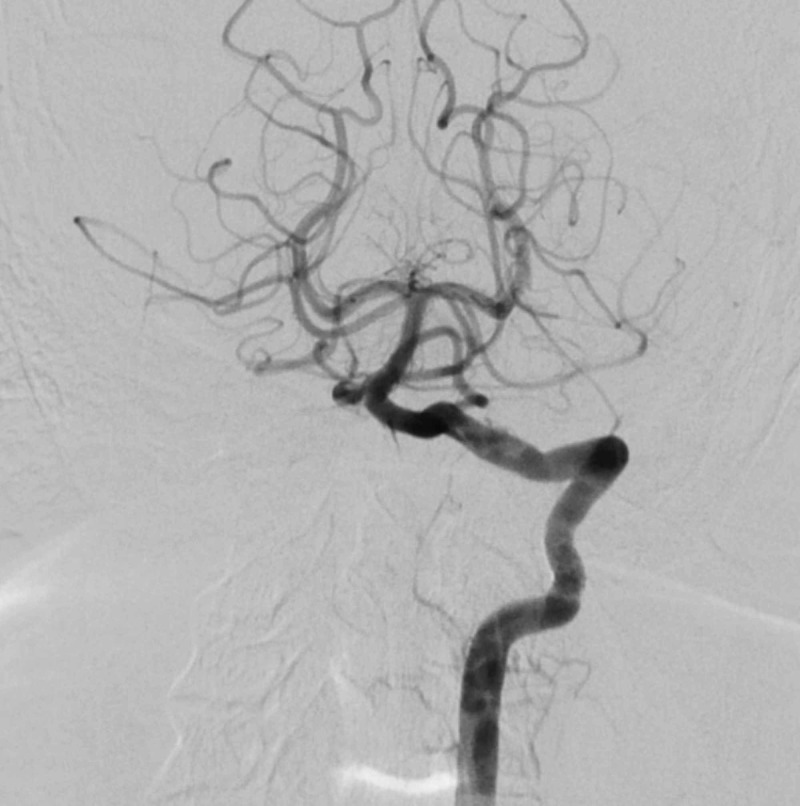
Check angiogram with left vertebral artery injection antero-posterior view at one month after the procedure shows normal anterograde filling of the posterior circulation through the left vertebral artery with complete exclusion of the pseudoaneurysm and the fistula from the left vertebral artery.

Following the procedure, the patient reported a gradual reduction in the size of the swelling with healing of the stab wound. At one month after the intervention, check angiogram showed patency of the stent with normal anterograde flow through the stent on selective left vertebral artery injection (Figure 5).

## Discussion

The treatment of vertebral arteriovenous fistula is necessary once the diagnosis is confirmed in both symptomatic and asymptomatic cases [2]. In symptomatic cases, the closure of fistula alleviates the symptoms such as tinnitus, headache and vertigo. Treatment is warranted even in the asymptomatic cases due to the fact that vertebral arteriovenous fistula can lead to spinal venous hypertension with myelopathic changes which may become irreversible over time if left untreated [2,3]. Surgical approaches to the treatment of vertebral arteriovenous fistula include ligature of the fistula site in cases of small punctate fistula and venous bypass grafting from subclavian artery to the distal vertebral artery in cases of complex fistula with multiple feeders. However, surgical techniques are associated with complications such as steal phenomenon, thrombo-embolic complications, haemorrhagic complications and injury to surrounding structures [1]. Endovascular techniques have become the first-line treatment option for vertebral arteriovenous fistula. Endovascular techniques may be broadly categorised into constructive and deconstructive techniques [3]. Constructive techniques include occlusion of the fistula using detachable balloon and/or coils while preserving the patency of the vertebral artery. Stents may also be used in some cases while maintaining the patency of the parent artery [4]. Deconstructive techniques include the occlusion of the fistula as well as the parent artery [2,3]. In our institute, we use the constructive endovascular techniques as far as possible. Deconstructive techniques may be used in those cases which pass the balloon occlusion test and have a good supply of the posterior circulation through the contralateral vertebral artery [1,2]. Our patient presented with a unique problem of a post-traumatic pseudoaneurysm associated with a vertebral arteriovenous fistula. An urgent endovascular treatment was warranted due to the risk of rupture of the pseudoaneurysm and possible complications due to mass effect and vertebral venous hypertension. On digitally subtracted angiogram, we could identify the site of the vascular injury at the V2 segment of left vertebral artery; however, the contrast was seen going into the pseudoaneurysm and the exact site of fistulous drainage into the vertebral venous plexus was not very clear. Among the options available, we opted for occlusion of the fistula using a covered stent across the site of the injury which excluded the pseudoaneurysm and the fistula from the left vertebral artery. This mitigated the steal of the blood flow from the left vertebral artery into the fistula and helped in restoration of the anterograde flow in the left vertebral artery. We faced a slight technical difficulty after deployment of the stent as the external diameter of the stent available at the time of the intervention was 3.5 mm and the calibre of the artery at the site of deployment was approximately 4 mm. Due to this discrepancy, we could see some residual filling of the pseudoaneurysm and the fistula immediately after the deployment of the stent. We decided to dilate the stent to approximate the vessel wall which ultimately led to complete occlusion of the pseudoaneurysm and the fistula. Since the intervention was done in an emergency setting, a loading dose of antiplatelets could not be given. We initiated intravenous tirofiban, which is a glycoprotein IIb/IIIa inhibitor, immediately after the deployment of the stent in order to prevent in-stent thrombosis and thromboembolic complications. Subsequently, the patent was put on dual antiplatelet treatment. Thus, we could achieve complete cure of a potentially life-threatening and disabling complication of traumatic vertebral artery injury within a reasonable cost for the patient.

## Conclusions

Vertebral arteriovenous fistula with associated pseudoaneurysm is a rare condition which requires treatment irrespective of patients symptoms. Endovascular placement of a covered stent can be used as a cost-effective therapeutic option in such cases, and a complete cure can be accomplished.

## References

[1] Briganti F, Tedeschi E, Leone G (2013). Endovascular treatment of vertebro-vertebral arteriovenous fistula. A report of three cases and literature review. Neuroradiol J.

[2] Aljobeh A, Sorenson TJ, Bortolotti C, Cloft H, Lanzino G (2019). Vertebral arteriovenous fistula: a review article. World Neurosurg.

[3] Felbaum D, Chidambaram S, Mason RB, Armonda RA, Liu AH (2016). Vertebral-venous fistula: an unusual cause for ocular symptoms mimicking a carotid cavernous fistula. J Neurointerv Surg.

[4] Rajadurai S, Muthukumaraswamy S, Hussain Z (2019). Endovascular treatment of vertebral-venous fistula with flow-diverting stent. World Neurosurg.

